# A Rare Observation: Forest Dormouse Occupying Nests of White‐Crowned Penduline Tit

**DOI:** 10.1002/ece3.71206

**Published:** 2025-03-30

**Authors:** Yu Huang, Hui Wang, Guobin Zheng, Piaopiao Ye, Wenqi Li, Kuerbanjiang Hanahati, Hongmei Liao, De Chen

**Affiliations:** ^1^ MOE Key Laboratory for Biodiversity Science and Ecological Engineering College of Life Sciences, Beijing Normal University Beijing China; ^2^ Advanced Institute of Natural Sciences, Beijing Normal University Zhuhai China; ^3^ Research Institute of Forestry, Ili Kazakh Autonomous Prefecture Yining China; ^4^ Agricultural Science Research Institute, Ili Kazakh Autonomous Prefecture Yining China

**Keywords:** breeding, natural nest, *Remiz*, rest, woven nest, Xinjiang

## Abstract

We report a novel observation of forest dormouse ( *Dryomys nitedula* ) using and modifying nests of the white‐crowned penduline tit ( *Remiz coronatus* ) as den and breeding sites in the Xinjiang Uyghur Autonomous Region, China. In the Huocheng Ili River Valley National Wetland Park, 9 out of 56 nests were occupied by dormice, with predation on nestlings observed in only one nest during the late breeding period. In contrast, at the Buergen Beaver National Nature Reserve, where penduline tits exhibit later breeding, four out of six occupied nests, from a total of 53 collected, were subject to predation, with either eggs or nestlings being preyed upon. These findings highlight the potential influence of temporal overlap between the breeding cycles of forest dormice and white‐crowned penduline tit, calling for more comprehensive research into the ecological consequences of this interaction under global change.

## Graphical abstract

We report the evidence of forest dormice using and modifying nests of the white‐crowned penduline tit for breeding in Xinjiang, China. Temporal overlap in breeding cycles influenced nest occupation, with predation observed more frequently in later‐breeding nests. These findings emphasize the need for further research into the ecological consequences of this novel interaction under global change.

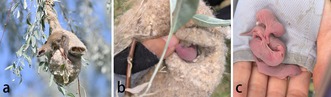

## 1 Introduction

Rodents (Rodentia), particularly dormice (Gliridae), rely heavily on the presence of old trees and nest boxes for both rest and breeding (Czeszczewik et al. 2008; Patel et al. 2021; Vinueza‐Hidalgo et al. 2024). Several dormice species compete with cavity‐nesting birds for limited cavity resources by taking over tree cavities or artificial nest boxes initially occupied by birds, leading to nest losses of breeding birds (Sarà et al. 2006; Juškaitis 2006; Adamík and Král 2008a). In most cases where dormice occupy the nests of open‐nesting birds, they are primarily observed preying on eggs or nestlings. Adamík and Weidinger (2024) documented direct predation evidence of the edible dormouse ( *Glis glis* ) on three species of open‐cup nesting songbirds. However, hazel dormice ( *Muscardinus avellanarius* ) have also been found to use abandoned open‐cup nests as a base for their summer nests (Berthold and Querner 1986; Fuchs 1987).

The forest dormouse ( *Dryomys nitedula* ) is an omnivorous, arboreal species known for building ball‐shaped nests with an exit in trees. This small rodent weighs between 36–61 g and has a head‐body length of 85–120 mm (Smith and Xie 2008). In addition to constructing its own nests, it frequently utilizes natural tree cavities and nest boxes, often favoring those already occupied by cavity‐nesting birds (Adamík and Král 2008a; Czeszczewik et al. 2008). Within these bird nests, the forest dormice have been observed preying on eggs, nestlings, or even adult birds (Juškaitis 2006). At times, forest dormice can be the key predators of certain cavity‐nesting birds. Wesołowski and Rowiński (2012) report that dormouse predation accounted for 31% of brood failure in blue tits ( *Cyanistes caeruleus* ). Besides, forest dormice have occasionally been observed occupying the nests of non‐cavity‐nesting birds, such as the dome‐shaped nests of wrens ( *Troglodytes troglodytes* ) and the cup‐shaped nests of blackbirds ( *Turdus merula* ) (Nevo and Amir 1964). However, most records primarily showed evidence of forest dormouse activities, such as eaten oak acorns, implying that dormice utilize these bird nests as feeding places rather than for breeding (Nevo and Amir 1964).

Penduline tits (*Remiz*) construct intricate, enclosed woven nests with a pouch‐like shape and small, tubular entrances. Unlike the simpler weaving found in species like wrens or blackbirds, penduline tit nests are meticulously crafted through a complex interlacing of plant fibers and animal hair, resulting in a secure and enclosed structure. These nests are suspended from branches, typically hanging on the weak terminal twigs of trees (Šustek et al. 1993). Their strategic placement and narrow entrance provide effective protection against large predators, with snakes posing the primary threat, followed by small mammals such as mongooses (Zheng et al. 2018; Lloyd et al. 2017). To attract females, unmated male penduline tits build a new nest for each breeding attempt, and once a pair is formed, both males and females collaborate to complete the nest (Hoi et al. 1994). Stubbe et al. (2012) recorded excrements of forest dormice in a few nests of penduline tits in Southwest Mongolia, but without further investigation.

In northwest China, the breeding grounds of the white‐crowned penduline tit ( *Remiz coronatus* ) coincide with the distribution range of the forest dormouse (Smith and Xie 2008; Wang et al. 2022). According to Stubbe et al. (2012), forest dormice in Southwest Mongolia (near the border with China) finish hibernation in the first days of May, with offspring born in the second half of June in tree nests. Meanwhile, the white‐crowned penduline tits migrate to their breeding grounds in April and breed from April to August, with their breeding season overlapping with that of the forest dormice.

In this study, we report that forest dormice regularly occupy the intricate hanging nests of the white‐crowned penduline tit, and for the first time, we observed breeding within these nests. We first observed this phenomenon during our 2023 survey and gathered more detailed data in 2024 to explore the potential impact of this interaction on the breeding success of the white‐crowned penduline tit.

## 2 Methods

**FIGURE 1:**
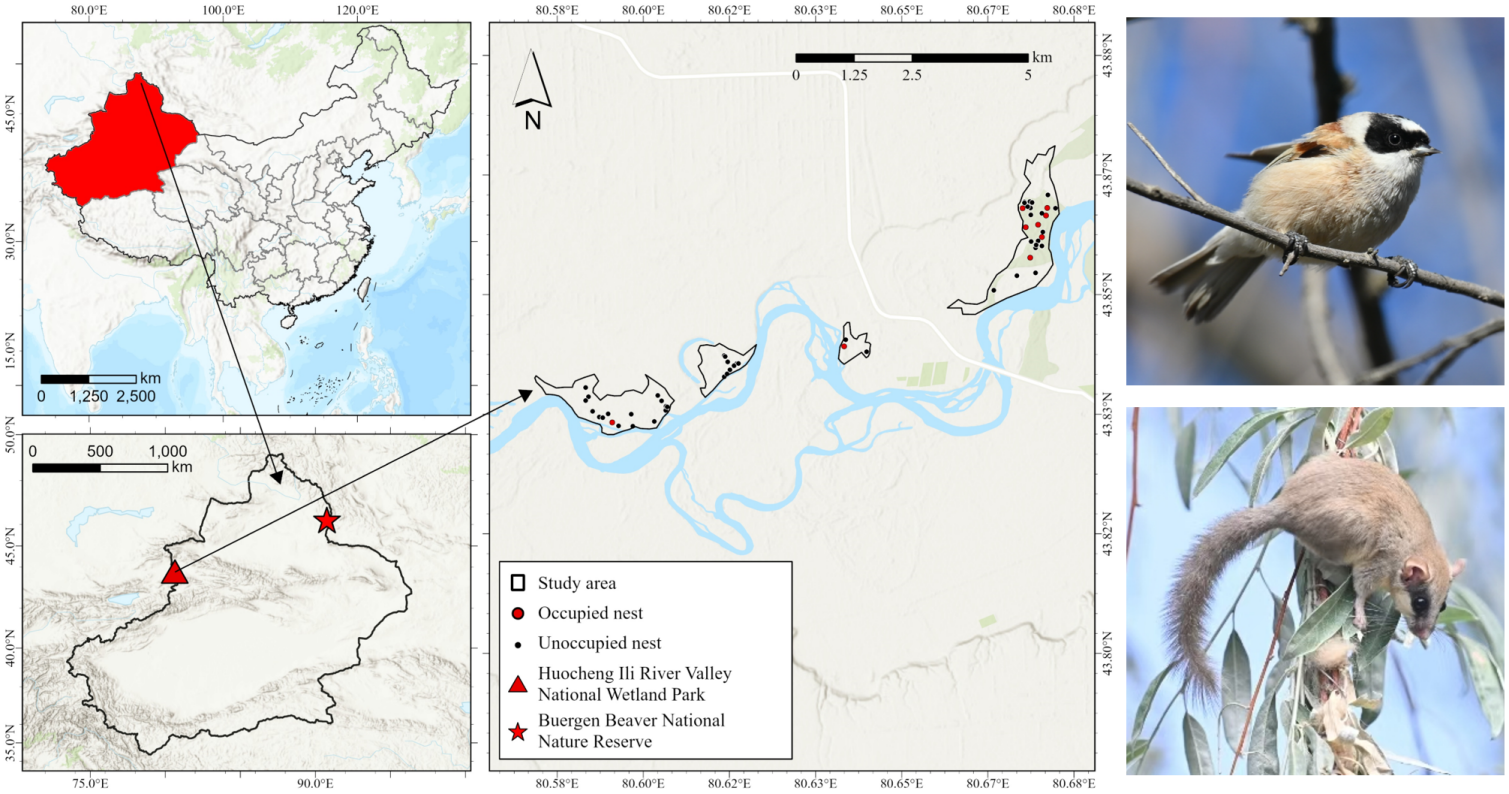
Study sites, nests distribution of white‐crowned penduline tits and species photos. The red triangle marks Huocheng Ili River Valley National Wetland Park (Ili), while the red star indicates Buergen Beaver National Nature Reserve (BG). Both sites are situated within Xinjiang Uyghur Autonomous Region, China (highlighted in red on the inset map of China). The right panel provides detailed the spatial distribution of collected white‐crowned penduline tit nests in Ili. Red dots indicate occupied nests, black dots unoccupied. Scale bars and coordinates are provided for spatial reference. Two species photos are shown on the right: Upper, white‐crowned penduline tit; lower, forest dormouse.

Fieldwork was conducted from April to August 2023 at the Huocheng Ili River Valley National Wetland Park (43°50′–43°52′ N, 80°35′–80°40′ E), and in 2024 at both the Huocheng Ili River Valley National Wetland Park and the Buergen Beaver National Nature Reserve (46°10′–46°13′ N, 90°46′–90°52′ E) both located in the Xinjiang Uyghur Autonomous Region, China (Figure 1). Hereafter, the Huocheng Ili River Valley National Wetland Park will be referred to as Ili, and the Buergen Beaver National Nature Reserve as BG. Both sites are located in inland river valley plains and belong to the temperate continental climate. Oleaster ( *Elaeagnus angustifolia* L.) and willow (*Salix*) are the dominant woody plants in the river valley forests of the Ili and BG study areas, respectively, and are also the primary nesting tree species for the white‐crowned penduline tit.

In 2023, we observed signs of forest dormice in the nests of white‐crowned penduline tits in Ili. After breeding ended in August, we inspected nests for signs of forest dormouse activity using a ladder. In 2024, we monitored the detailed breeding ecology of white‐crowned penduline tits. Each nest was checked every 2–3 days, ensuring that key reproductive events such as pairing, egg‐laying, hatching, fledging and abandonment were not missed; such monitoring continued until the 16th day of nestlings. After most white‐crowned penduline tits completed breeding in August, nests were collected to examine potential signs of forest dormouse activity. Before collecting, an endoscope was used to confirm that no animal was present inside the nests. The nests were then removed from the trees using a pole pruner, marked with a nest ID, and brought back to the laboratory for further inspection and measurements.

The droppings of forest dormouse were used to determine whether the collected nest has been occupied. If eggshell fragments or nestling remains were found, they were considered predation by the forest dormouse, as snakes, the primary predators of the penduline tit, do not leave such traces, and other potential predators, like raptors, do not maintain the nest structure so intact.

Detailed measurements of nest parameters were recorded in Ili, including nest height, nest width, nest bottom thickness, and nest volume. Nest height was measured as the maximum length (±0.5 cm) from the top to the bottom. Nest volume was determined by filling the nest with fine sand and transferring it to a 500 cm^3^ graduated cylinder (±1 cm^3^). Finally, the nest was vertically cut in half, and the bottom thickness was measured with a sliding caliper (±1 mm) (Szentirmai et al. 2005). The normality of these nest parameter data was tested using the Shapiro–Wilk test in R version 4.1.3 (R Core Team 2021). For normally distributed data, *T*‐tests were conducted to compare the characteristics of occupied and unoccupied nests, while for non‐normally distributed data, the Wilcoxon rank sum test was used. Additionally, the spatial distribution of occupied and unoccupied nests in Ili was mapped using ArcGIS Pro version 3.1.6 (Esri 2023). The area and forest cover proportion of the 4 study patches in Ili (Figures 1 and S1) were also calculated, with land cover data sourced from Gong et al. (2019).

## 3 Results

**FIGURE 2:**
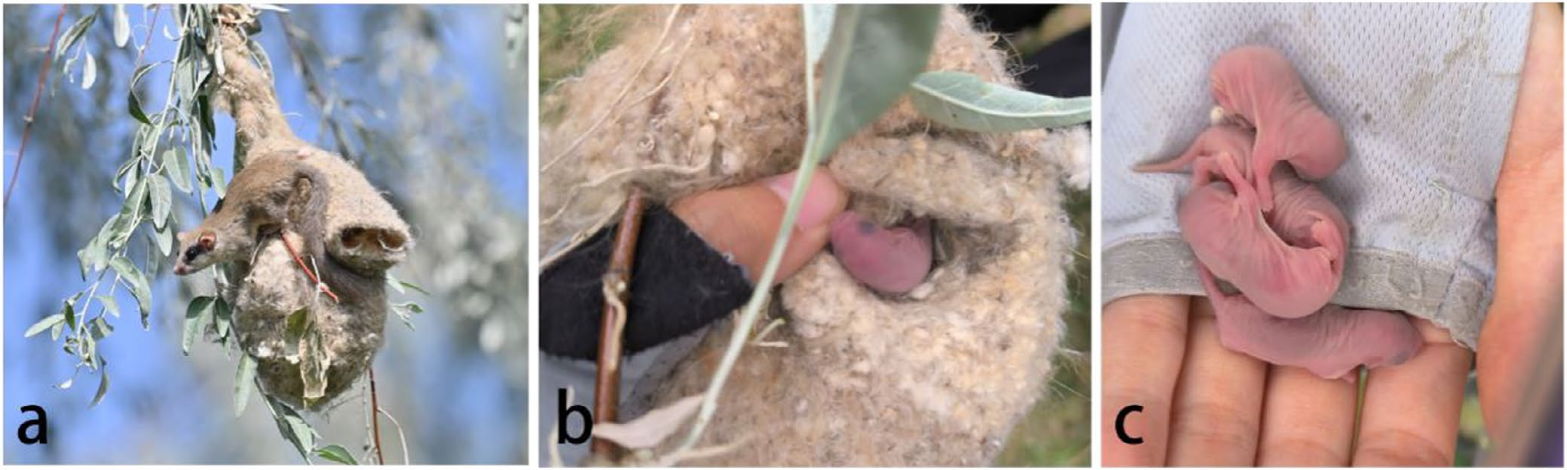
Dormouse adult and offspring found in the nests of white‐crowned penduline tit in 2023 in Ili (a) Dormouse adult coming out of the nest, (b, c) Dormouse offspring found in the nest.

The breeding seasons of the white‐crowned penduline tit in Ili and BG, defined as the period from the first nest observed date to the last fledging date in our study area, were observed to span from April 10th to August 15th and from May 5th to July 30th, respectively. In 2023, we checked 32 nests in Ili, of which 8 showed signs of dormice activity. Among these, 7 nests contained dormouse feces, while one nest harbored an adult dormouse and 4 juveniles (Figure 2), providing direct evidence of reproduction.

**FIGURE 3:**
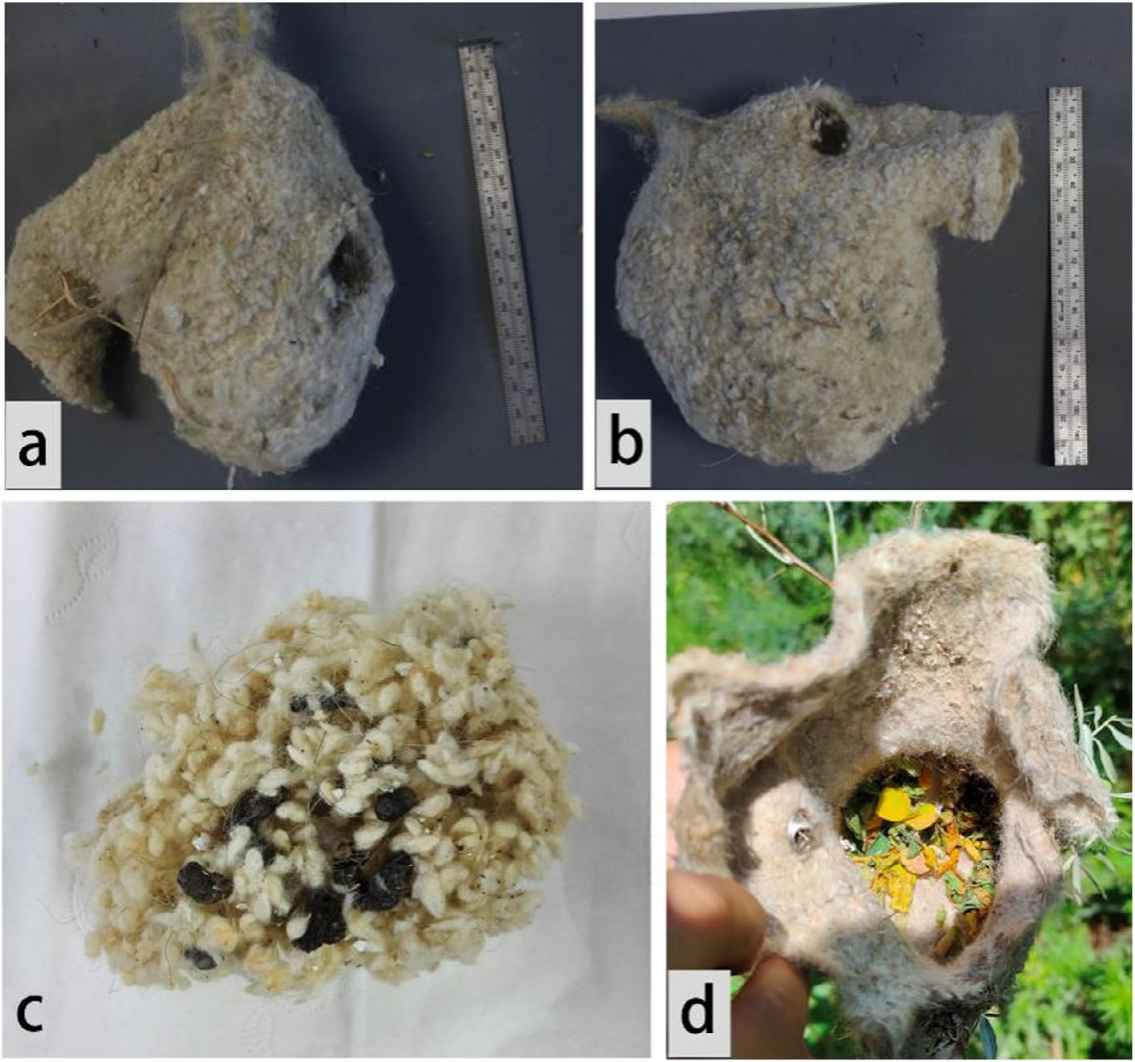
Modification of occupied nests of white‐crowned penduline tits by forest dormice (a, b) Hole made by forest dormice at top or rear, (c) Interior filled with shredded nesting materials, (d) Interior filled with leaves.

A total of 56 nests were collected from Ili between August 3rd and 5th, 2024. Of these, 9 showed signs of dormice activity. Additionally, 53 nests were inspected in BG between August 1st and 2nd, of which 6 were occupied, with 1 containing an adult dormouse and the other 5 showing only traces of dormice. In a significant proportion of cases (60%, 9 out of 15 nests occupied), forest dormice were observed to create a small hole at the top or rear of the nest (Figure 3a,b). Two primary modifications were observed within the nests (Table 1). The first modification involved the loosening of tightly packed nesting material into small clumps (Figure 3c and Modified interior I in Table 1), making the entire nest softer. The second modification involved the addition of leaves to the nest (Figure 3d and Modified interior II in Table 1), which were only found in Ili.

**TABLE 1:** Breeding and modification information of occupied nests in 2024.

Region	NestID	Modification		Laying date	Breeding success
		Hole	Modified interior		
Ili	Ili24027	At the rear	I	2024/5/4	Success
Ili	Ili24019	No hole	II	2024/5/9	Success
Ili	Ili24045	At the top	I	2024/5/14	Success
Ili	Ili24049	At the rear	I	2024/5/11	Success
Ili	Ili24084	No hole	I	2024/5/11	Success
Ili	Ili24083	At the rear	I	2024/5/16	Nest was abandoned during incubation
Ili	Ili24053	No hole	II	2024/5/18	Success
Ili	Ili24121	No hole	II	2024/5/25	Success
Ili	Ili24114	No hole	I	2024/6/11	Nestlings were preyed on
BG	BG24032	At the rear	I		Nest was abandoned before egg‐laying
BG	BG24093	At the top	I		Nest was abandoned before egg‐laying
BG	BG24040	At the rear	I	2024/5/30	Nestlings were preyed on
BG	BG24092	No hole	I	2024/6/8	Eggs were preyed on
BG	BG24077	At the rear	I	2024/6/9	Nestlings were preyed on
BG	BG24104	At the rear	I	2024/6/12	Nestlings were preyed on

**FIGURE 4:**
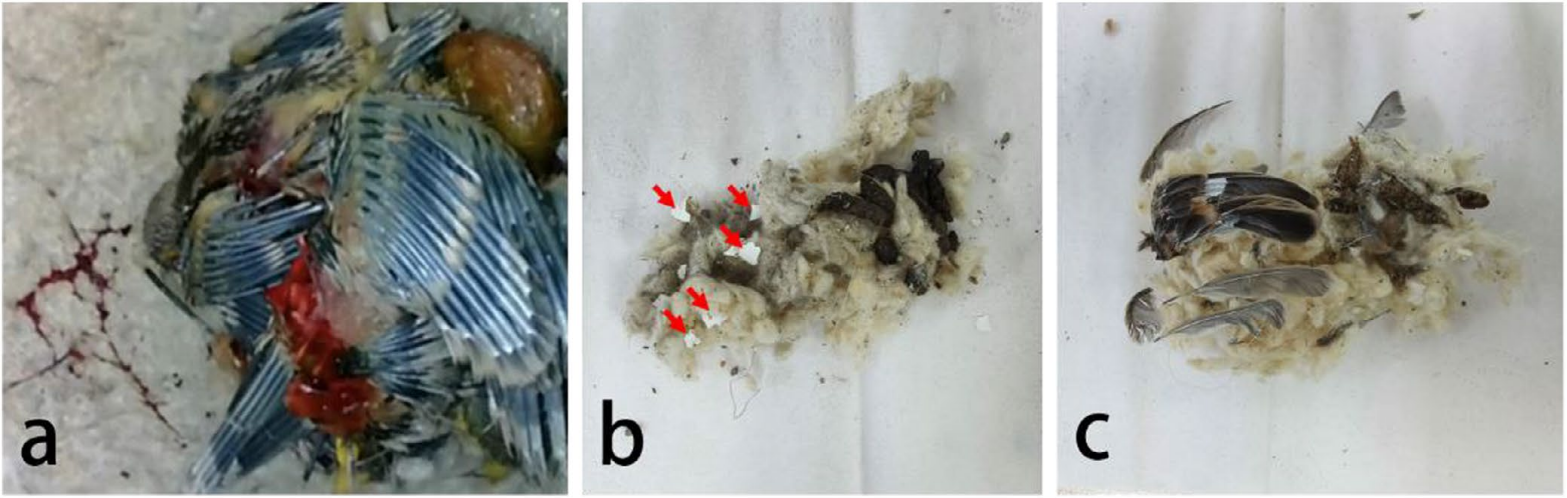
Evidence of predation in occupied nests of white‐crowned penduline tits: (a) Chewed nestlings, (b) White eggshell fragments (indicated by the red arrows), (c) Remnants of nestlings.

Among the nine occupied nests in Ili in 2024, one was abandoned by white‐crowned penduline tits during incubation, while seven were successfully monitored until the nestlings reached 16 days—a stage typically indicative of breeding success. The egg‐laying dates for these 8 nests ranged from May 4th to May 25th (Table 1). However, 1 nest with a later egg‐laying date (June 11th) showed signs of dormice activity, including evidence of nestlings being chewed (Figure 4a). In BG, white‐crowned penduline tits initiated their breeding cycle much later in the season. Of the six occupied nests, two were abandoned before egg‐laying, while the remaining four showed signs of predation on eggs or nestlings (Figure 4b,c). Interestingly, the egg‐laying dates for these 4 nests were concentrated in early June (Table 1), similar to the depredated nest in Ili.

To further explore potential habitat preferences and nest selection of forest dormouse, we analyzed the spatial distribution of occupied nests in Ili in 2024. We calculated the areas of the four study regions from left to right (Figure 1) as 0.98 km^2^, 0.36 km^2^, 0.17 km^2^, and 1.26 km^2^, with corresponding forest cover proportions of 3.87%, 3.47%, 2.35%, and 9.04%. Among the nine occupied nests, seven were located within the largest of the four monitored patches, while the remaining two occupied nests were located in two smaller patches (Figure 1). The occupied nest density in the forest areas for the four patches is 0.04, 0, 0.14, and 0.50 individual per square kilometer, respectively.

We also compared several parameters between occupied and unoccupied nests (Table 2) to determine whether occupied nests had unique structural characteristics. No significant differences were found in any of these parameters.

**TABLE 2:** Comparison of parameters between occupied and unoccupied nests.

	Nest height (mm)	Nest width (mm)	Nest volume (cm^3^)	Nest bottom thickness (mm)
Occupied nests	143.67 ± 7.52	78.00 ± 4.58	256.00 ± 23.39	35.16 ± 5.63
(*N* = 9)	(*N* = 9)	(*N* = 9)	(*N* = 3)	(*N* = 8)
Unoccupied nests	140.91 ± 11.19	78.43 ± 5.80	225.78 ± 36.09	39.64 ± 11.35
(*N* = 47)	(*N* = 46)	(*N* = 46)	(*N* = 41)	(*N* = 44)
Test value	*t*(15.91) = −0.92	*t*(13.55) = −0.25	*W* = 25.5	*W* = 228
*p*	0.37	0.81	0.10	0.19

## 4 Discussion

In previous studies, forest dormice were often found to utilize the natural tree cavities or nest boxes already occupied by cavity‐nesting birds (Adamík and Král 2008a; Czeszczewik et al. 2008). Our study expands on these findings, revealing that forest dormice also commonly occupy and modify the woven nests of white‐crowned penduline tits. Unlike natural tree cavities or nest boxes, the nests of the penduline tit are intricate, enclosed woven structures that hang from tree branches, making them potentially more difficult to access. In fact, the nest predation rates by mammals are relatively low due to the strength and complexity of the nests (Lloyd et al. 2017; Zheng et al. 2018; Persson and Öhrström 1989). The sturdy, well‐insulated structure and relatively concealed location of the penduline tit nests make them ideal refuges or breeding sites for forest dormice, especially when natural tree cavities are scarce. Forest dormice made several modifications to the nests of white‐crowned penduline tits to meet their specific needs (Figure 3), demonstrating their high adaptability in using bird nests. The creation of holes at the top or rear of the nests likely served functional purposes, such as providing access or escape routes. Additionally, the modification of the nesting material likely enhanced the comfort and insulation of the nest, and the addition of leaves is consistent with the nesting material used in natural forest dormouse nests (Nevo and Amir 1964). The modifications suggest that the dormice were tailoring the nest to suit their own environmental and physiological needs. While no direct evidence of breeding was found in 2024, we discovered dormouse adults and offspring in one nest in 2023 (Figure 2), suggesting that dormice use these nests as breeding sites, likely due to limited availability of other suitable locations. This marks the first instance of forest dormice breeding in naturally bird nests.

Regarding nest selection, there is no significant difference between occupied and unoccupied nests in terms of structural parameters, suggesting that forest dormice did not show a preference for specific nest traits. However, the concentration of occupied nests in the largest patch raises the possibility that larger forest areas may provide more favorable conditions for forest dormice. A well‐developed and dense understory, which enhances structural connectivity, has been identified as an important factor for characterizing suitable nest sites for this species (Juškaitis et al. 2012; Fedyń et al. 2021; Magomedov 2015; Pilāts et al. 2012). Future studies could further explore whether larger forest patches indeed offer denser understory and greater resource availability, making them more suitable habitats for dormice than smaller, simpler patches.

Our findings also indicate that the forest dormice may affect late‐breeding birds, potentially posing notable risks to their reproductive success. In our two study sites, the breeding times of the white‐crowned penduline tits differed, leading to varying degrees of temporal overlap with dormice activity. In the earlier‐breeding population of Ili, the breeding peak is staggered with the dormice's active period, resulting in almost no predation, except for one later‐breeding nest (Table 1). In contrast, in the later‐breeding population of BG, the high overlap with dormice activity results in 67% of eggs and nestlings being predated. These findings align with Adamík and Král (2008a), who reported that migratory species such as *Ficedula albicollis* experience higher predation rates than resident species due to delayed breeding, which overlaps with dormice activity. Furthermore, within the context of global climate change, rising spring temperatures have led to the earlier termination of hibernation in the edible dormice ( *Glis glis* )—common predator of birds. This shift has led to an increasing overlap in nest box usage between dormice and birds (Adamík and Král 2008b), highlighting the need for further research on the ecological consequences of these changes.

In conclusion, our study underscores the complex interactions between forest dormice and white‐crowned penduline tits. We demonstrate that dormice can occupy and modify woven bird nests, highlighting their adaptability in utilizing alternative nesting sites. Furthermore, our findings emphasize the significant impact of temporal overlap between dormice activity and bird breeding periods. These results call for further research into the ecological dynamics of nest site competition and predator–prey interactions, particularly in light of the ongoing effects of climate change.

## Author Contributions

**Yu Huang:** formal analysis (lead), investigation (equal), writing – original draft (lead), writing – review and editing (equal). **Hui Wang:** investigation (equal), methodology (supporting), writing – review and editing (equal). **Guobin Zheng:** investigation (supporting), writing – review and editing (supporting). **Piaopiao Ye:** investigation (supporting). **Wenqi Li:** investigation (supporting). **Kuerbanjiang Hanahati:** investigation (supporting). **Hongmei Liao:** investigation (supporting). **De Chen:** conceptualization (lead), project administration (lead), writing – review and editing (supporting).

## Conflicts of Interest

The authors declare no conflicts of interest.

## Supporting information


Appendix S1.

## Data Availability

This article provides descriptive information so all available data are provided in photo format within the main document. The nest structure data and R code used in this study are publicly available at: https://github.com/Huangyu146/A‐Rare‐Observation‐Forest‐Dormouse‐Occupying‐Nests‐of‐White‐crowned‐Penduline‐Tit.
