# Supplementary material for: A Rare Observation: Forest Dormouse Occupying Nests of White‐Crowned Penduline Tit
Source: Ecol Evol. 2025 Mar 30;15(4):e71206. doi: 10.1002/ece3.71206 (PMC11955254; doi:10.1002/ece3.71206)
Supplement: Supplementary file 1 — Appendix S1. [file ECE3-15-e71206-s001.docx]

# Supporting Information


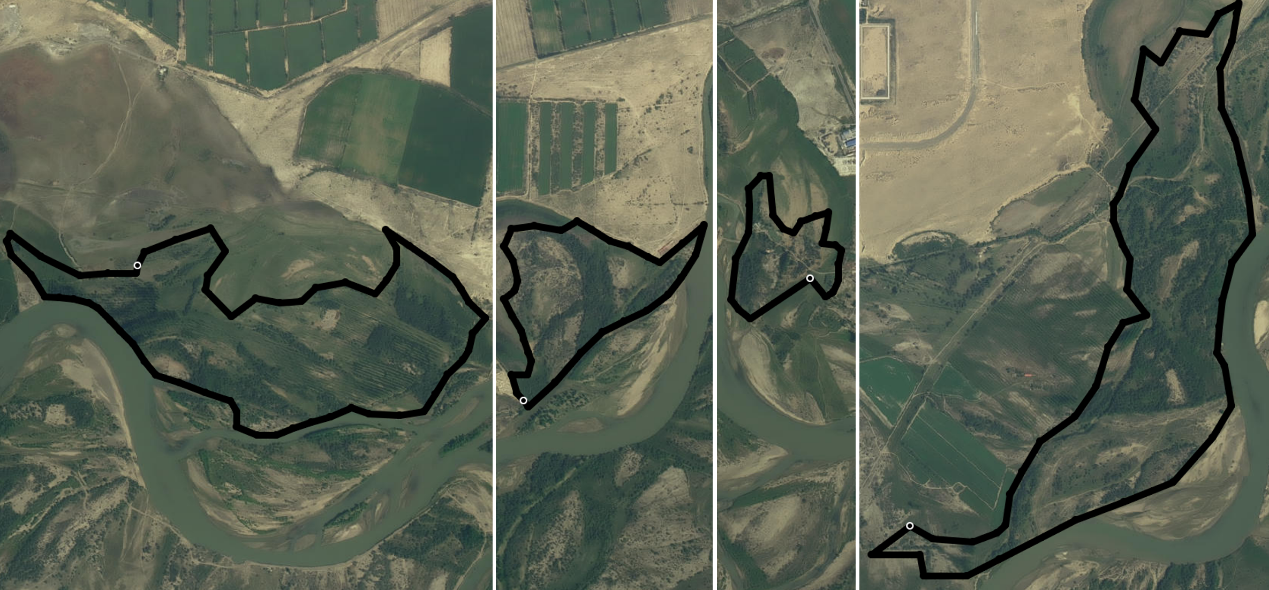


Fingure S1 Satellite images of the four study areas in Ili with the black line indicating the boundaries.
